# Development of a Novel Piezoelectric Sensing System for Pavement Dynamic Load Identification

**DOI:** 10.3390/s19214668

**Published:** 2019-10-28

**Authors:** Qian Zhao, Linbing Wang, Kang Zhao, Hailu Yang

**Affiliations:** 1 National Center for Material Service Safety, University of Science and Technology Beijing, Beijing 100083, China; zhaoqian928@xs.ustb.edu.cn (Q.Z.); zhaok666999@163.com (K.Z.); 2 Joint USTB-Virginia Tech Lab on Multifunctional Materials, USTB, Beijing 100083, China; 3 Department of Civil and Environmental Engineering, Virginia Tech, Blacksburg, VA 24061, USA

**Keywords:** piezoelectric sensors, weigh-in-motion, lateral position detection, finite element simulation

## Abstract

In order to control the adverse effect of vehicles overloading infrastructure and traffic safety, weight-in-motion (WIM)-related research has drawn growing attention. To address the high cost of current piezoelectric sensors in installation and maintenance, a study on developing a low-cost piezoceramic sensing system is presented in this paper. The proposed system features distributed monitoring and integrated packaging, for calculating vehicle’s dynamic load and its wheel position. Results from the laboratory tests show that the total output of the sensing system increases linearly with the increase of the peak load when the loading amplitude is 5–25 kN (equivalent to the half-axis load of 20–100 kN); when the loading frequency is between 15 Hz and 19 Hz (equivalent to a speed of 17.8–23.2 km/h), the total output of the system fluctuates around a value of 1.305 V. Combined with finite-element simulation, the system can locate load lateral position with a resolution of 120 mm. Due to the protection packaging, the peak load transferred to the sensing units is approximately 4.36% of the applied peak load. The study indicates the proposed system can provide a promising low-cost, reliable and practical alternative for current WIM systems.

## 1. Introduction

Nowadays, overloading of heavy vehicles has been a great concern when it comes to infrastructures and traffic [1]: it significantly reduces the service life of roads and bridges, increases the workload and expense of maintenance and reconstruction, lowers traffic efficiency, and raises the risk of traffic accidents. Fortunately, as studies have shown [2,3], weight-in-motion (WIM) systems have a significant effect on limiting overloading, by strengthening the supervision and hence punishment of overloaded vehicles.

However, both of the characteristics of the sensors and the complicated vehicle–pavement interaction, have restricted the measurement accuracy and long-term stability of the WIM systems. The sensor errors come from electrical noise, non-linearity, temperature sensitivity, and loading duration and rate effects, etc. [4], while the vehicle–pavement interaction contributes to dynamic errors in weighing, effected by the pavement state and vehicles’ driving situation [5]. Thus, the recent research is mainly focused on the two areas [6]: design of good-performance sensors and control of disturbing factors from the vehicle–pavement interaction.

For different WIM systems, the sensing technologies include the bending plate, the load cell, piezoelectric sensors, optic fibers [7] and capacitive sensors [8]. Among them, piezoelectric sensors are sensitive, stable, and feasible for installation, and are widely applied and the focus of constant research attention. Due to the characteristics of the piezoelectric effect, this kind of sensors are quite suitable for a high-speed WIM (HS-WIM) system, a trend in future applications.

Commonly, there are three kinds of piezoelectric sensors: the piezo-quartz, the piezopolymer and the piezoceramic. As a natural piezoelectric material, the piezo-quartz is the most sensitive, expensive and fragile among them, typically applied as the sensitive part in an I-steel structure WIM system. The measurement accuracy of the piezo-quartz depends significantly on the pavement surface evenness and the pavement structure, resulting in a high cost in installation and regular calibration. Therefore, the piezo-quartz WIM system is less considered in remote regions of developing countries. With nearly one third price of the piezo-quartz sensor [9], the piezopolymer sensor is often used as piezoelectric cable in the pre-selection of overloading vehicles and traffic monitoring. Due to its high temperature dependency [10], it is difficult to calibrate for highly accurate weighing results. Moreover, the flexible design of cables makes it vulnerable to pavement rutting and potholes. The piezoceramic, represented by lead zirconate titanate (PZT), has gradually shown its potential in a WIM system. It has the advantages of structural simplicity, low cost, quick response, high reliability and good structural compatibility. Over the past few decades, sensors based on the PZT materials have been popular in structural health monitoring [11,12] and pavement energy harvesting [13,14,15].

Recently, Sirous Alavi et al. [16] confirmed the good performance of piezoceramic sensors and piezo-polymer sensors in both Portland cement concrete and asphalt concrete (AC) pavements in controlled field tests, after nearly 3.2 million ESALs (Equivalent Single Axle Load) and 183,000 ESALs respectively. The low loading speed was of 5 km/h, which made it difficult for data tuning and further conclusions. Gangbin Song et al. [11] proposed a piezoceramic-based smart aggregate for applications in concrete civil structures. Shuang Hou et al. [17] developed a load-monitoring method for an AC pavement by using SA (Smart Aggregate), demonstrated its linear sensitivity to the applied load. However, when embedded in the real pavement at a depth of 10 cm, only 4/15 of the SA survived under a concrete mixing truck’s load (with an axle load up to 17.5 t) [18]. Furthermore, Guofeng Du [19] et al. interpreted the output performance of SA under external loading by finite element models. Xiaoming Yang et al. [20] fabricated a cement-based piezoceramic sensor and evaluated its frequency response and linearity under complex load, square load and random load. Shanglin Song et al. [21] provided an aggregate-shape piezoceramic sensor with the help of a 3D printed mold, proving its promising application in vehicle weighing. Considering the application in unsupervised traffic, Haocheng Xiong and Yinning Zhang [22] presented a prototype of a piezoelectric WIM (P-WIM) system consisting of nine piezoceramic disks encapsulated as a whole, concluded that the P-WIM performed reliably with a cost of 80% less than the permanent WIM.

Unfortunately, the current research mainly focuses on the development of the single sensor and its performance in lab tests. When applied in a complex loading situation in the field, the single sensor may be insufficient in strength and have limited accuracy. A design containing several sensing parts can provide enough strength while the relationship of output and layout of inserted sensors should be investigated. Moreover, the piezoceramic can be distributed separately in the direction perpendicular to traffic moving, which is also a useful feature [23] ignored in the past. Some research in bridge WIM (B-WIM) systems started considering the transverse position (TP) of vehicles [24]. Shizhi Chen et al. [25,26] developed a WIM system by LG FBG (Long Gauge Fiber Bragg Grating) sensors and tested the performance under the situation of two vehicles with different transverse position by small-scale experiments. Yang Yu et al. [27] proposed an algorithm for B-WIM based on weighing sensors at different girders to detect the TP of vehicles, proved to be promising for improving weighing accuracy. Unlike the lateral consistency of piezo-quartz and piezopolymer cable, the individual piezoceramic sensors can provide wheel lateral location for wandering monitoring.

Therefore, this study designed a piezoceramic sensing system for pavement WIM, containing four sensing units to calculate a vehicle’s dynamic load and its wheel position. Through laboratory experiments, the system was tested under sinusoidal loading of different amplitudes and frequencies, ranging from 5 to 25 kN and 5 to 19 Hz. Then, a finite-element model of the sensing system was built to simulate three kinds of loading conditions: linearly uniform loading, sinusoidal loading and equivalent wheel loading. Based on the non-uniformly distributed wheel loading simulation, an algorithm of load lateral location has been introduced. From the results of theoretical calculation, laboratory tests and simulations, the proposed system was testified to be a promising low-cost, reliable and practical alternative for current WIM systems.

## 2. Methodology

### 2.1. Principle of the Piezoelectric Transducer

When being applied an external force along the polarization direction, the piezoelectric material forms a potential difference at the same direction. It is known as the piezoelectric effect, which can be expressed as Equation (1) [28]:

(1) $$D=d\sigma +\lambda^{\sigma }E,$$

where:

- *D*—electric displacement vector, C/m^2^;
- *d*—piezoelectric strain constant matrix, C/N;
- *σ*—stress vector, Pa;
- *λ^σ^*—dielectric constant matrix when the stress is constant, F/m;
- *E*—external electric field intensity, N/C.

**Figure 1:**
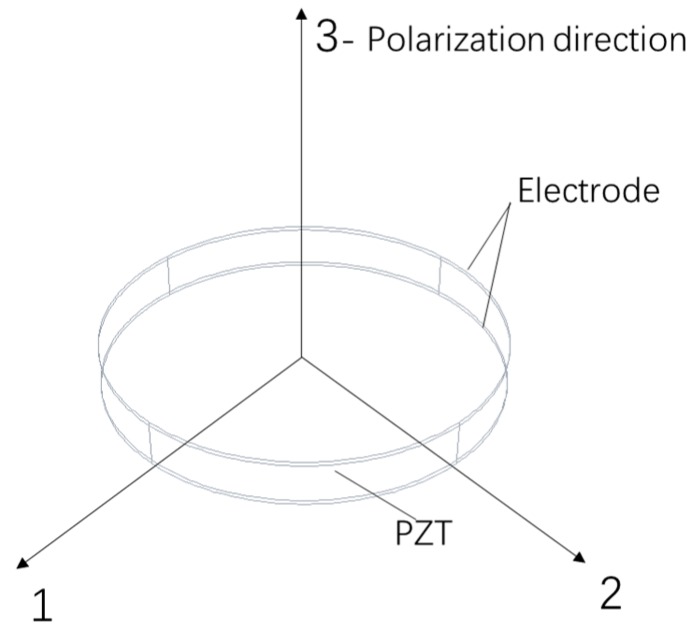
The circular piezoelectric patch of *d*_33_ mode.

In this study, a circular piezoceramic patch (mentioned as ‘the PZT patch’ in the following text) of *d*_33_ mode works as the sensing part, as shown in Figure 1, and the 3-direction is the polarization direction. Since there is no external electric field at both sides of the PZT patch, Equation (1) can be simplified as Equation (2):

(2) $$\left[\begin{array}{c} D_{x}\\ D_{y}\\ D_{z} \end{array}\right]=\left[\begin{array}{cccccc} 0 & 0 & 0 & 0 & d_{15} & 0\\ 0 & 0 & 0 & d_{24} & 0 & 0\\ d_{13} & d_{23} & d_{33} & 0 & 0 & 0 \end{array}\right]\left[\begin{array}{c} \sigma_{xx}\\ \sigma_{yy}\\ \sigma_{zz}\\ \sigma_{yz}\\ \sigma_{zx}\\ \sigma_{xy} \end{array}\right],$$

where:

- $D_{i}$—electric displacement in direction of i, C/m^2^;
- $d_{ij}$—piezoelectric strain constant in direction normal to i,j, C/N;
- $\sigma_{ij}$—stress component in direction normal to i,j, Pa.

The generated charge is the integral of the electric displacement in the corresponding area. Due to the shape of the PZT patch, the areas in the direction 1 and 2 are much smaller than that in direction 3, which can be negligible, so the total charge can be written as Equation (3) [11].

(3) $$q=D_{z}A_{z},$$

Among them:

- q—the total generated charge, C;
- *A_z_* is the area of the PZT patch in the 3-direction (as shown in Figure 1), m^2^.

Assuming that the external force is mainly in the 3-direction (i.e., the polarization direction), Equation (3) can be simplified as Equation (4):

(4) $$q=d_{33}\sigma_{zz}A_{z},$$

The relationship between the output voltage of the PZT patch and the charge is as Equation (5):

(5) $$V=q/C_{p}$$

where *C_p_* is the internal capacitance of the piezoceramic, F. *C_p_* can be as in Equation (6):

(6) $$C_{p}=\frac{\varepsilon_{r}\varepsilon_{0}A_{z}}{h},$$

where *ε_r_* is the relative dielectric constant, dimension is 1; *ε*_0_ is the vacuum dielectric constant, F/m; h is the thickness of the PZT patch, m.

According to the literature [19], when the PZT patch is subjected to a dynamic load, the ratio of the output voltage (*V*) to the load (*σ*) can be defined as the sensitivity *S*, as in Equation (7):

(7) $$S=\frac{V}{\sigma }.$$

As the PZT patch is assumed to be only subjected to axial load, i.e., *σ_zz_* = *σ*, Equation (7) can be written as Equation (8):

(8) $$S=\frac{d_{33}h}{\varepsilon_{r}\varepsilon_{0}}.$$

Since the piezoelectric constant *d*_33_ and the internal capacitance *C_p_* can be directly measured by the instrument in this research, Equation (8) can be expressed as Equation (9) to calculate the theoretical sensitivity:

(9) $$S=\frac{d_{33}A_{z}}{C_{p}}.$$

### 2.2. Design of the Sensing System

Piezoceramic is a polycrystalline inorganic material with good piezoelectricity, high dielectric constant and high rigidity. When it is used as a sensor, its main failure modes include fatigue cracking under high-frequency vibration, overloading caused by stress concentration, or electrode failure of corrosion due to water (vapor) or solutions [29]. In order to cope with the complicated and harsh environment of the road, the structure and packaging design of the PZT patch are required to ensure its sensing performance and durability. Metal packaging performs well in anti-deformation, which can provide enough protection for integrated sensing units, while organic materials are of low density, good toughness, corrosion resistance and easy processing. Therefore, they have been widely used in recent years in engineering applications [30,31]. Thus, the packaging design in this study consists of two parts: the metal packaging of a single piezoelectric patch and the entire packaging of the piezoelectric array of organic material.

#### 2.2.1. Design of the Piezoelectric Sensing Unit

**Figure 2:**
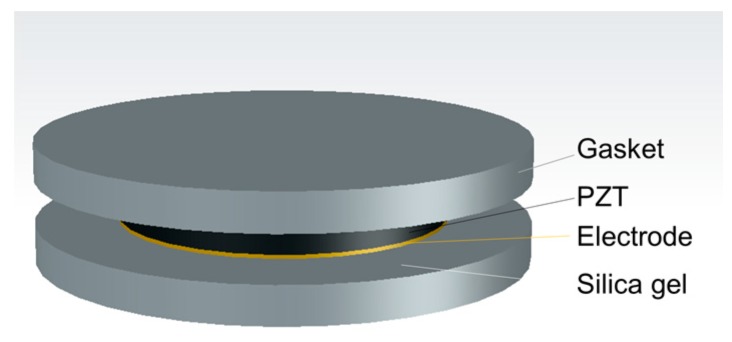
Schematic of the piezoelectric sensing unit.

The piezoelectric sensing unit is composed of four parts: a piece of piezoelectric patch, electrode sheets, stainless steel gaskets, and electronic silica gel as shown in Figure 2. The size of the PZT patch is φ20 × 2 mm, and the electrode sheets are of φ20 × 0.2 mm. With a size of φ30 × 1.5 mm, the gaskets are a little bigger than the PZT patch, which can leave silica gel to fill the side of the PZT patch. The flexible electronic silica gel can prevent the piezoelectric material and the electrode from water or vapor effectively, as well as the side force. Combining with the stainless-steel gaskets, the gel can ensure the PZT patch stressed in the polarization direction as uniformly as possible. The piezoceramics used in this research is PZT-4 produced by Baoding Hongsheng Acoustic Electronic Equipment Co., Ltd. The material parameters of PZT-4 are shown in Table 1.

In theory, the sensitivity of the piezoelectric sheet to the load is expressed as $S_{t}$. According to the component size parameter used in the experiment and the average value of the performance parameters in Table 1, the sensitivity calculation Equation (10) is deduced from Equation (9):

(10) $$S_{t}=0.3069\;V/\mathrm{MPa}$$

#### 2.2.2. The Packaging Design of the Piezoelectric Array

**Figure 3:**
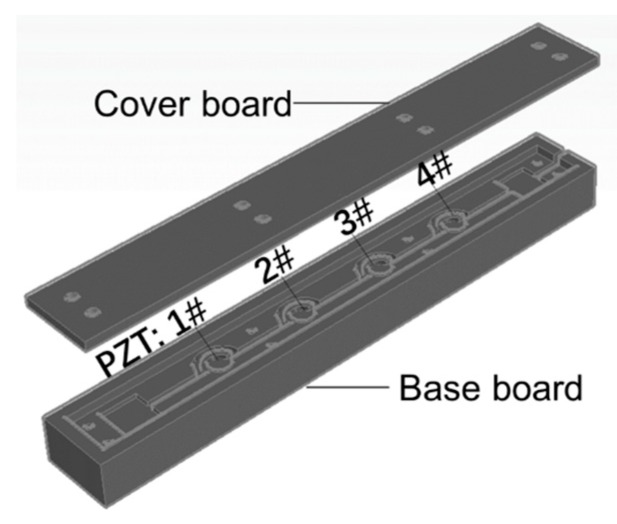
The structure of the piezoelectric array system.

In order to capture the wheel load and its lateral position, a strip-shaped piezoelectric array containing four piezoelectric units is designed. Instead of the small-scale individual piezoceramic sensors, the system can provide enough protection for the sensing parts. There are three parts in this system: the base, the sensing units with wire, and the cover board, as presented in Figure 3. Under the cover board, the four units are able to work in collaboration. As assumed in Section 2.1, the external force on the sensing unit is mainly in the 3-direction (as shown in Figure 1, that is the vertical direction). Thus, the interval of four units should be less than the width of a tire, which ranges from 175 to 335 mm. For heavy trucks with dual tires, the total length should be able to cover the width of two tires at least. Considering the structural stability under overloading, the overall size of the system is set to be 750 mm × 100 mm × 60 mm (L × W × H), and the units are deployed by an interval of 120 mm in the middle of the system. The cover board and the base board are both made of glass fiber-reinforced nylon.

## 3. Fabrication of Sensing System, Experiment and Simulation

### 3.1. System Fabrication

**Figure 4:**
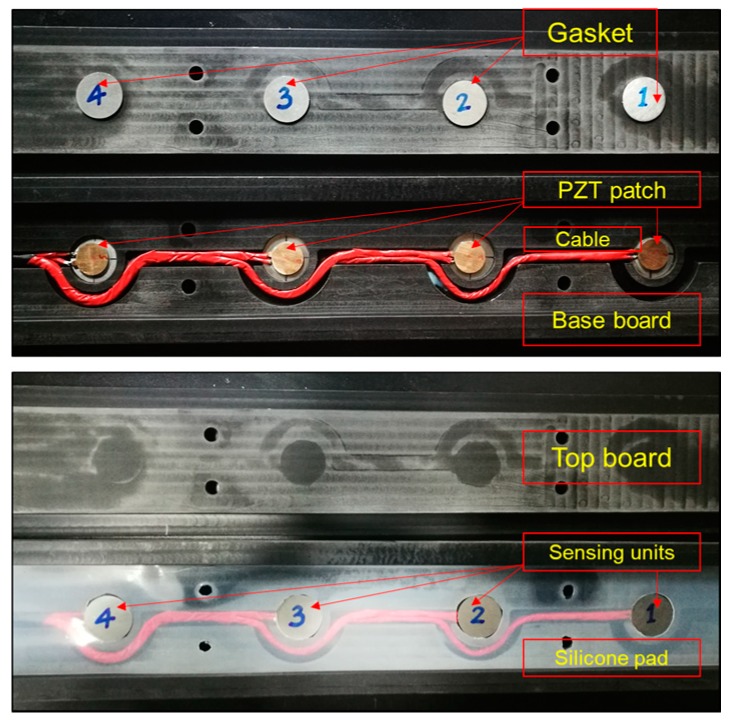
Internal structure of the sensing system.

Based on the design in Section 2.2, the sensing units were fabricated by the following steps: (1) welding of the PZT patch with the shielded wire; (2) marking the center of the bottom gaskets; (3) positioning and bonding of the PZT patches with the bottom gaskets; (4) filling the sides of the PZT patches with flexible electronic silica gel; (5) positioning and bonding of the upper gasket. Subsequently, the prepared units were placed in an orderly fashion and bonded in the base board, as well as the shielded wire. Then, the gap of the wire groove was filled with silicone rubber and the waterproof silicone pad was laid. Finally, with the cover board mounted, bolts were installed and it was sealed tightly. The internal structure of the system is shown in Figure 4.

### 3.2. Laboratory Experiments

#### 3.2.1. The Test Arrangement

**Figure 5:**
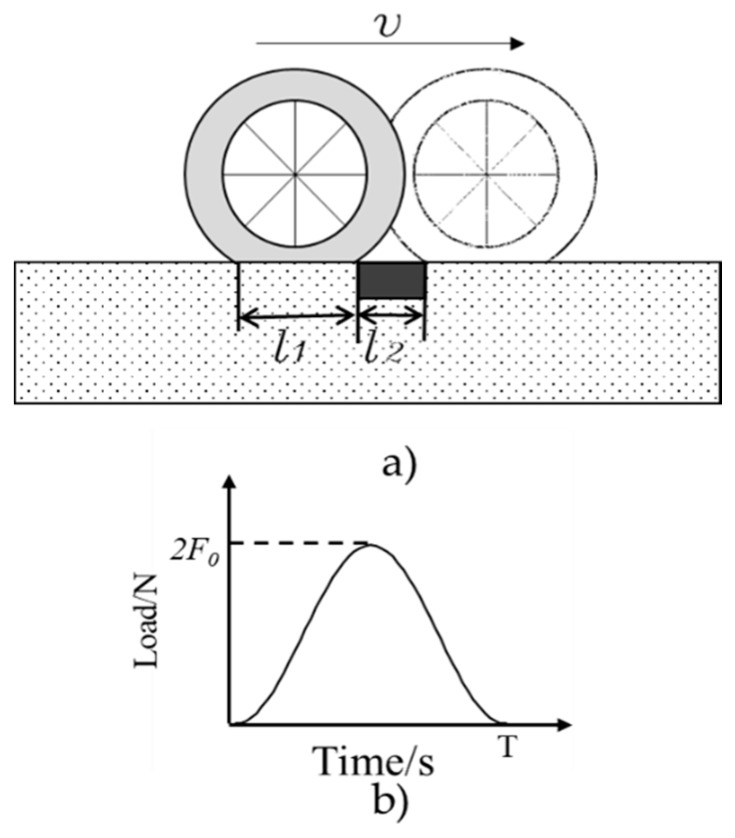
The simplified process of a vehicle loading on the embedded system. (**a**) wheel travelling along the pavement with a speed of v; (**b**) the equivalent semi-sine loading curve.

When a vehicle is traveling on the pavement, the stress experienced by the road surface can be simplified to a semi-sine wave load [17,21]. The process of the vehicle loading on the embedded WIM system can be simplified as Figure 5.

Then, the loading frequency ($f_{l}$) caused by vehicle can be defined by Equation (11):

(11) $$f_{l}=\frac{1}{T}=\frac{v}{l_{1}+l_{2}}$$

where:

- T—loading period, s;
- v—speed of the vehicle, m/s;
- $l_{1}$—length of the wheel-pavement contacting area, m;
- $l_{2}$—width of the embedded sensing system, m.

Thus, to investigate the sensing system’s performance under dynamic loads in the pavement, it was necessary to carry out amplitude scanning and frequency scanning experiment. These experiments aimed at determining the relationship between the output of the sensing system and the input load and the relationship of the output of the sensing system with the loading frequency.

**Figure 6:**
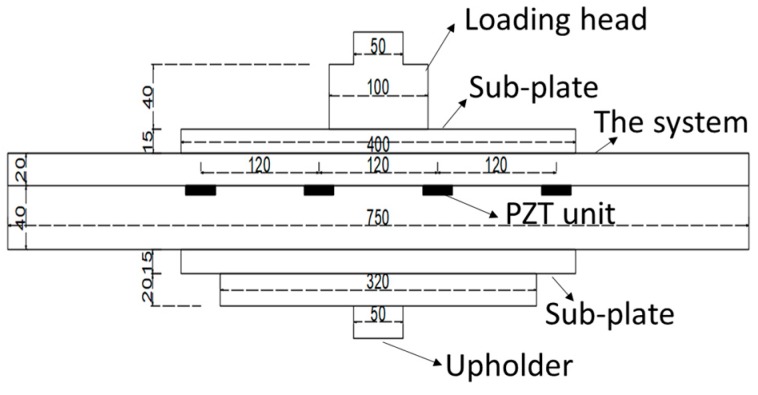
Schematic diagram of the loading structure.

An electro-hydraulic servo machine (MTS-370.10) was adopted in the experiment, with a maximum load of 100 kN. A loading head for compressive testing was used to apply vertical force, and the heading area is a circle with a diameter of 100 mm. The system was of 750 mm × 100 mm × 60 mm (L × W × H), placed right in the middle. When embedded in the pavement, the system is assumed to be tightly connected to the bottom and bounded to the four sides. Since the system is designed to undertake the wheel loading of different sizes, the heading area is not wide enough to test the sensing units’ performance. Considering the loading stability, two stainless steel plates (with a size of 400 mm × 100 mm × 15 mm (L × W × H)) served as sub-plates to ensure that each unit can be loaded. The loading structure is shown in Figure 6. All of the piezoelectric units were located 20 mm beneath the cover board and in the loading zone between two sub-plates.

#### 3.2.2. Loading Procedures

**Figure 7:**
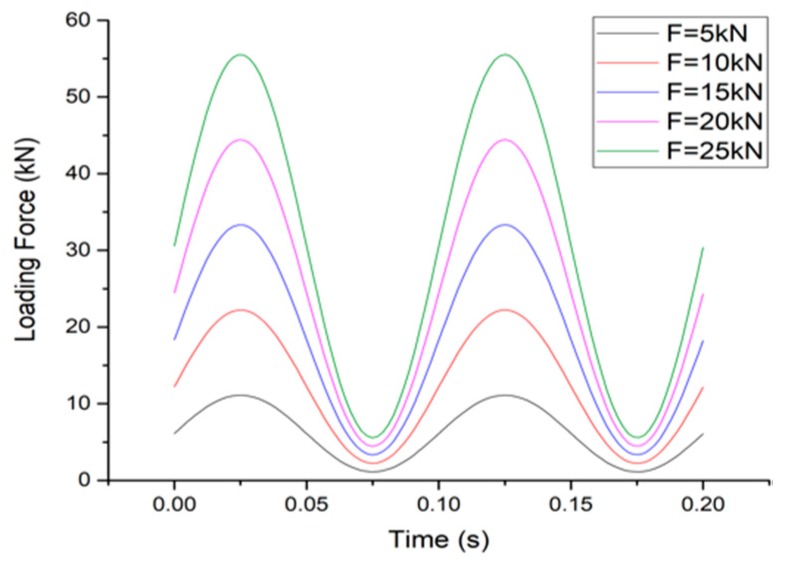
Amplitude scanning loading curve.

According to the standard axle load specified in China’s current road design specifications (100 kN) and the loading range of the machine, the amplitude scanning experiment contains loading amplitude of 5, 10, 15, 20, 25 kN, equivalent to the total load of 10–50 kN. Given that the width of the system is nearly half of a typical tire–pavement contact zone, the experiment can represent a half-axis load of 20–100 kN, which can cover the 200% overloading of the standard situation. With a frequency of 10 Hz, the amplitude scanning loading scheme is as shown in Figure 7.

**Figure 8:**
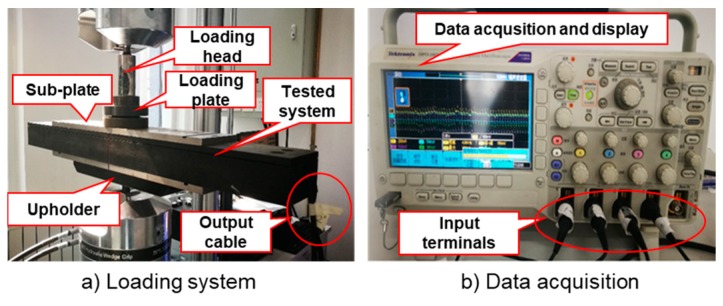
Indoor test process.

From Figure 5a and Equation (11), the loading frequency is related to the vehicle’s speed and the length of the tire–pavement contact area. As introduced in literature [32,33,34], the length of the tire–pavement contact area ($l_{1}$) is usually taken from 0.22 m to 0.24 m due to different loading conditions. In Section 2.2, $l_{2}$ is the width of the designed system, which is 0.1 m. Due to the stability of the loading machine, the loading frequency is set from 5 to 19 Hz, with an interval of 2 Hz, which covers a speed of 1.6 to 6.46 m/s (that is 5.76–23.3 km/h). The loading amplitude is set as 10 kN. The loading system and data acquisition system are shown in Figure 8.

### 3.3. Finite-Element Simulation

**Figure 9:**
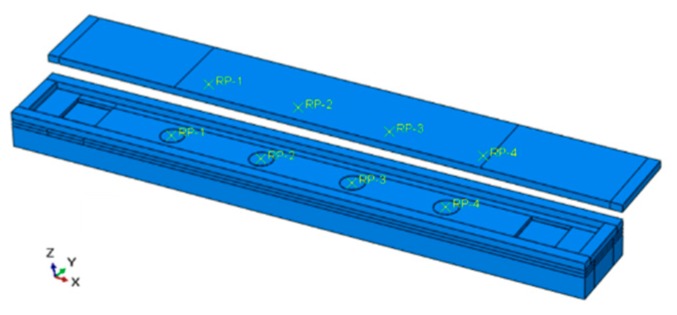
The piezoelectric sensing system model.

Combining with the above experiments, the software ABAQUS 6.14 was adopted to simulate the stress distribution and piezoelectric performance of the sensing system. For simplification, the sensing unit was considered to be a cylinder made of piezoelectric materials in the three-dimensional model. Ignoring the effect of wires, the whole structure consisted of three parts: the cover board, the piezoelectric units and the base board. For simplification, the material of the cover and the base board was assumed as having isotropic linear elasticity [14,35] and the element type of these two parts was C3D8R (three-dimensional reduced integrated solid element with eight nodes). The material parameters are shown in Table 2 [36]. The piezoceramic material (PZT-4) was orthogonal isotropic, interpreted by the stiffness matrix ($c_{e}$, Equation (12)), the piezoelectric constant matrix ($e_{T}$, Equation (13)) and the dielectric constant matrix ($\varepsilon_{T}$, Equation (14)), with an element type of C3D8E (three-dimensional electrical solid element with eight nodes) [19]. For each piezoelectric unit, the electric potential of the bottom surface was set to zero, while the top surface was set to an equipotential surface. All of the components inside the system were constrained as ‘Tie’ and the finite element model is shown in Figure 9.

(12) $$c_{e}=\left[\begin{array}{cccccc} 126 & 77.8 & 74.3 & 0 & 0 & 0\\ 77.8 & 126 & 74.3 & 0 & 0 & 0\\ 74.3 & 74.3 & 115 & 0 & 0 & 0\\ 0 & 0 & 0 & 24.1 & 0 & 0\\ 0 & 0 & 0 & 0 & 25.6 & 0\\ 0 & 0 & 0 & 0 & 0 & 25.6 \end{array}\right]\times 10^{3}\;\mathrm{MPa}$$

(13) $$e_{T}=\left[\begin{array}{ccc} 0 & 0 & -5.2\\ 0 & 0 & -5.2\\ 0 & 0 & 15.1\\ 0 & 0 & 0\\ 12.7 & 0 & 0\\ 0 & 12.7 & 0 \end{array}\right]\;N/V\cdot m$$

(14) $$\varepsilon_{T}=\left[\begin{array}{ccc} 6.464e-9 & 0 & 0\\ 0 & 6.464e-9 & 0\\ 0 & 0 & 5.622e-9 \end{array}\right]\;C/m$$

#### 3.3.1. Linearly Uniform Loading

**Figure 10:**
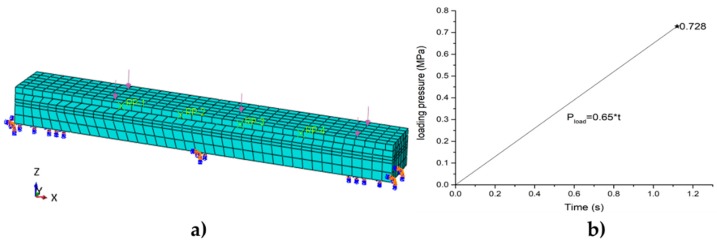
Linearly uniform loading. (**a**) Load and boundary conditions; (**b**) load curve.

The linearly uniform loading condition and load curve are shown in Figure 10. The bottom was fully constrained and the top surface was the loading surface. The maximum loading stress was 0.728 MPa.

#### 3.3.2. Sinusoidal Loading Based on the Lab Experiment

**Figure 11:**
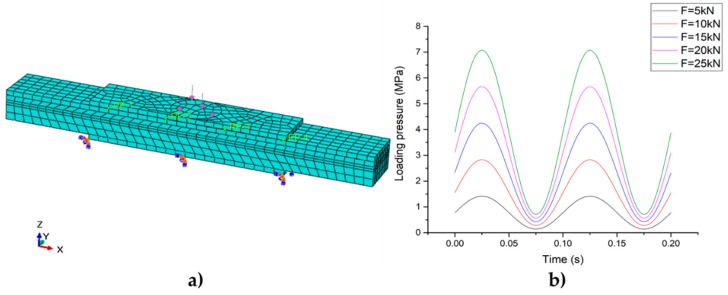
Sinusoidal loading. (**a**) Load and boundary conditions; (**b**) load curve.

The boundary conditions and the sinusoidal load curve of the test are shown in Figure 11. The bottom of the sub-plate-2 was restrained, and the center of the sub-plate-1 was pressed. The loading zone was the same size as the loading head area in the lab experiment.

#### 3.3.3. Equivalent Wheel Loading at Different Lateral Positions

**Figure 12:**
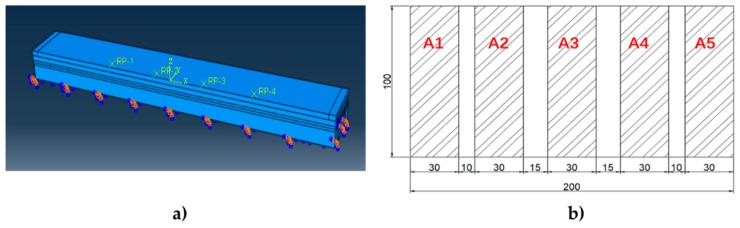
Equivalent wheel loading. (**a**) Load and boundary conditions; (**b**) load distribution.

Commonly, the lateral position of vehicles wandering, especially of heavy vehicles, is an important factor in surveying pavement distress and in traffic monitoring [37]. Based on the piezoelectric effect and the structure of the sensing system, it can be assumed that the system is able to locate the lateral position of the wheel through the sensing units. Hence, the simulation of the equivalent wheel load acting on different lateral positions of the sensing system was carried out. Figure 12a shows the load and boundary conditions. According to the actual width of the structure, the wheel load acting on the system was taken as a rectangle of 200 mm × 100 mm. Considering the tread spacing, the rectangular was divided into five zones, named A1, A2, A3, A4 and A5, as shown in Figure 12b. Due to the non-uniform distribution of tire stress, the stress ratio of each loading zone was A1:A2:A3:A4:A5 = 0.5:0.9:1:0.9:0.5 and the stress amplitude of A3 was 0.7 MPa. The loading center acted respectively on the Left (the connection center of 1# and 2#PZT), the Middle (the connection center of 2# and 3#PZT) and the Right (the connection center of 3# and 4#PZT). The load was applied by the subroutine of DLOAD.

## 4. Results and Discussion

### 4.1. Simulation of Linearly Uniformly Distributed Loading

**Figure 13:**
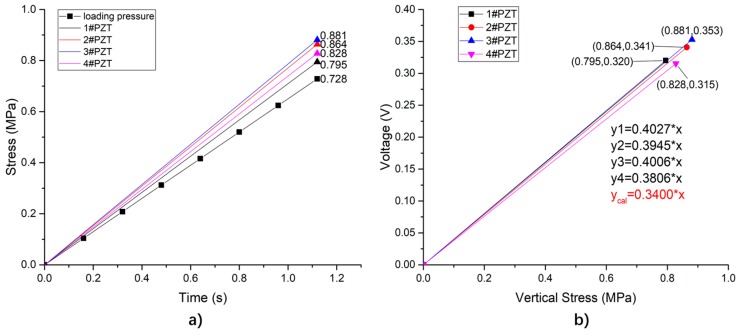
The piezoelectric sensing units under uniform load. (**a**) Vertical stress of each piezoelectric element; (**b**) comparison of analog sensitivity and theoretical sensitivity of each piezoelectric element.

When the sensing system is subjected to a linearly uniform load at the top surface, the vertical stress of each unit is shown as Figure 13a, and the corresponding output of the four units are presented in Figure 13b. According to the definition of sensitivity (as Equation (9)), the theoretical sensitivity of the simulation is calculated to 40 be 0.34 V/MPa, due to the equivalent size of the piezoelectric unit. From Figure 13b, the average simulated sensitivity of different units is 0.3946 V/MPa, with a standard deviation of 0.01. Compared with the theoretical sensitivity of the simulation, the average simulated sensitivity is 16% higher.

According to the simulation results, the load taken by the PZT patches ($load_{1})$ can be calculated as Equation (15), and the total load applied on the system ($load_{2}$) as Equation (16):

(15) $$load_{1}=\left(\sigma_{1}+\sigma_{2}+\sigma_{3}+\sigma_{4}\right)*A_{pzts}$$

(16) $$load_{2}=\sigma_{0}*A_{0}$$

where:

- $\sigma_{1}$—the stress of 1#PZT, which is 0.795 MPa in this situation;
- $\sigma_{2}$—the stress of 2#PZT, with a value of 0.864 MPa;
- $\sigma_{3}$—the stress of 3#PZT, which is equal to 0.881 MPa;
- $\sigma_{4}$—the stress of 4#PZT, of 0.828 MPa in this situation;
- $A_{pzts}$—the top surface area of the PZT patch;
- $\sigma_{0}$—the stress of the top surface of the system, as 0.728 MPa;
- $A_{0}$—the top surface area of the sensing system.

After calculation, the rate of $load_{1}$/$load_{2}$ equals to 4.36%. Thus, under the protection of the system package structure, the load on the sensing unit can be 4.36% of the overall load of the system, indicating that the system can fully protect sensitive components and avoid premature failure.

### 4.2. Tests and Simulation Results of Sinusoidal Loading

#### 4.2.1. Experimental Results

Amplitude scanning loading

**Figure 14:**
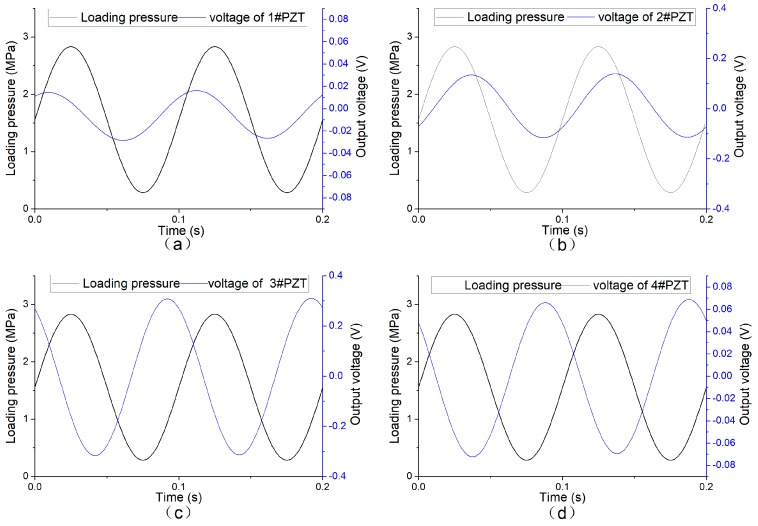
Output voltage of each channel of the system when loading frequency 10 Hz loading amplitude 10 kN. (**a**) 1#PZT output signal; (**b**) 2#PZT output signal; (**c**) 3#PZT output signal; (**d**) 4#PZT output signal.

When applied a load with an amplitude of 10 kN and a frequency of 10 Hz, the sensing system produced a series of voltage signals, acquired by the oscilloscope with a low-pass filter, as shown in Figure 14. As shown in the figure, all of the four channels have a stable signal output, and the output frequency is consistent with the loading frequency. Due to the different distance to the center of the load, there are different phase differences. According to the output amplitude, the peak output of the 2# and 3# piezoelectric units is about 3–6 times that of the 1# and 4# units. In accordance with the loading condition shown in Figure 8a, that is, the pressure center is at the connection center of 2# and 3# piezoelectric units.

**Figure 15:**
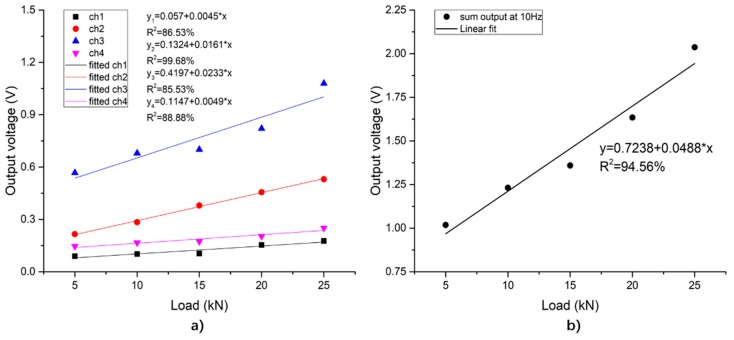
Relationship between the peak-to-valley value of the output voltage of the system with different loading amplitudes. (**a**) The output of each piezoelectric unit; (**b**) the total output of the sensing system.

When the loading frequency keeps at 10 Hz, the output voltage of the sensing system increases linearly with the growing load amplitude, as shown in Figure 15. Figure 15a shows the output signals of the piezoelectric units, and Figure 15b shows the sum of voltages of the sensing system. From Figure 15a, due to the symmetry, the slopes of 1#PZT and 4#PZT are extremely close. The slopes of 2#PZT and 3#PZT are, respectively, 0.0161 and 0.0233, are of the same magnitude and significantly larger than those of 1#PZT and 4#PZT, which suits the fact that the loading center is between 2#PZT and 3#PZT. Therefore, it is possible to locate the position of the load according to the output signals of the piezoelectric units. According to Figure 15b, as the loading frequency and the loading position are both fixed, the total output voltage of the system increases linearly with the load amplitude. Therefore, the amplitude of the load can be monitored effectively according to the total output of the system.

#### 4.2.2. Frequency Sweep Sinusoidal Loading Experimental Results

**Figure 16:**
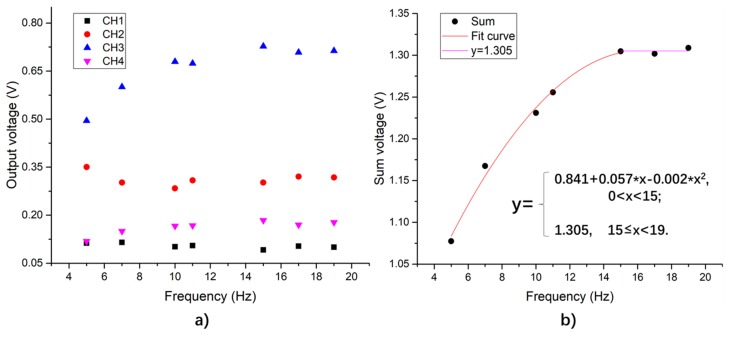
Output voltage peak-to-valley value vs. load frequency when loading amplitude is 10 kN. (**a**) Output of each piezoelectric unit; (**b**) piezoelectric sensing system total output.

When the loading amplitude is 10 kN, the output of the system under different frequencies are as shown in Figure 16. From Figure 16a, as the loading frequency increases, the output signals of the individual piezoelectric units vary from each other, while the trend of the total output is more obvious: when the loading frequency is lower than 16 Hz, the total output of the system increases as the frequency increases; when the loading frequency is between 15 Hz and 19 Hz, the total output of the system fluctuates around a value of 1.305 V. Since the piezoceramic material is a kind of dielectric material, the internal capacitance and leakage resistors of the PZT patch can form a RC circuit (with resistance and capacitance). The output voltage of the RC circuit is affected by the loading frequency. When external frequency is much lower than the resonant frequency of the PZT materials (the resonant frequency is usually several kHz or MHz), the output voltage gradually increases and peaks near a stable value as the frequency increases.

#### 4.2.3. Simulation Results of Sinusoidal Loading Finite Element

**Figure 17:**
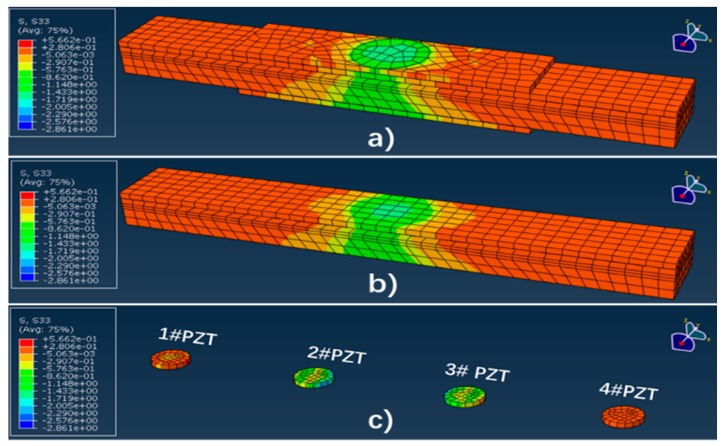
The stress distribution of the system when the equivalent load amplitude is 10 kN. (**a**) Load stress distribution; (**b**) system stress distribution; (**c**) piezoelectric single stress distribution.

**Figure 18:**
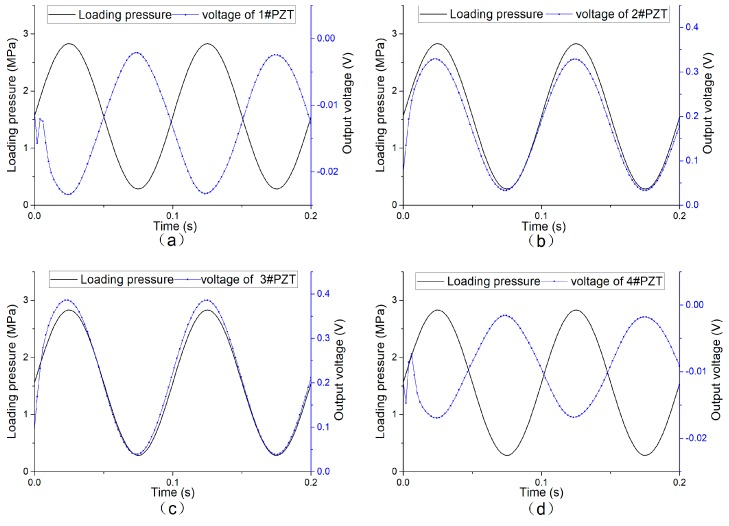
Simulated voltage output of each piezoelectric unit under sinusoidal loading. (**a**) output of 1#PZT; (**b**) output of 2#PZT; (**c**) output of 3#PZT; (**d**) output of 4#PZT.

According to the finite element model established in 3.2, the stress distribution and the output voltage of the system under different loads are obtained. As a comparison with the experiment results, Figure 17 and Figure 18 are the simulation results of the system under the load with an amplitude of 10 kN and a frequency of 10 Hz.

**Figure 19:**
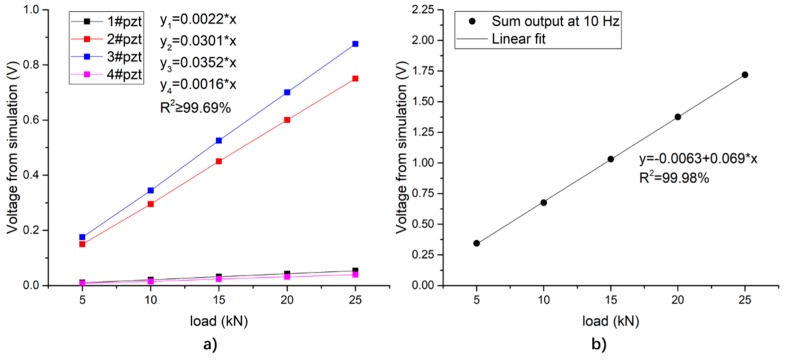
Relationship between the peak-to-valley value of the output voltage and the peak value of the load under sinusoidal loading simulation. (**a**) The output of each piezoelectric unit; (**b**) the total output of the piezoelectric sensing system.

As the peak load increases, the peak-to-valley voltage of each piezoelectric unit changes as shown in Figure 19a, showing good linearity. Due to the different distance from the loading center, the output values of the 1# and 4# piezoelectric units are much lower than those of the 2# and 3# piezoelectric units. Because of the difference in meshing, the output values of the units are approximately symmetrical, but not identically. Figure 19b shows the variation of the total output of the system with the loading amplitude.

#### 4.2.4. Comparison Results of the Experiment, Simulation and Theoretical Calculation

**Figure 20:**
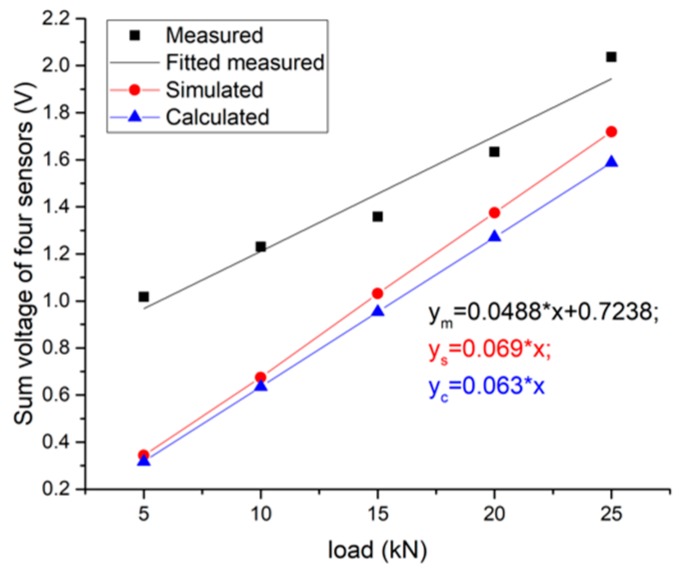
Comparison of measured, simulated and calculated values of total system output at 10 Hz loading frequency.

The comparison is made based on the sinusoidal loading at a frequency of 10 Hz. The measured value is the linear fitting value of the average peak value of the system under different loading amplitudes in the loading test (as in Figure 14b); the simulated value is the average value of the peak-to-valley voltage of the system in the simulation (as in Figure 18b); the calculated value is obtained from the vertical stress of the piezoelectric units, substituted into the stress-voltage sensitivity Equation (10), as interpreted in Figure 20.

Since the actual piezoelectric units are prestressed by the packaging with bolts, the system’s output is not zero when the external load is zero, which is different from the simulation/theory results. The slope, representing the sensitivity, is the key of the comparison. From the results in Figure 20, taken the theoretical calculation as the basis, the error of the measured sensitivity is −23%, and the error of the analog sensitivity is 8.6%.

**Figure 21:**
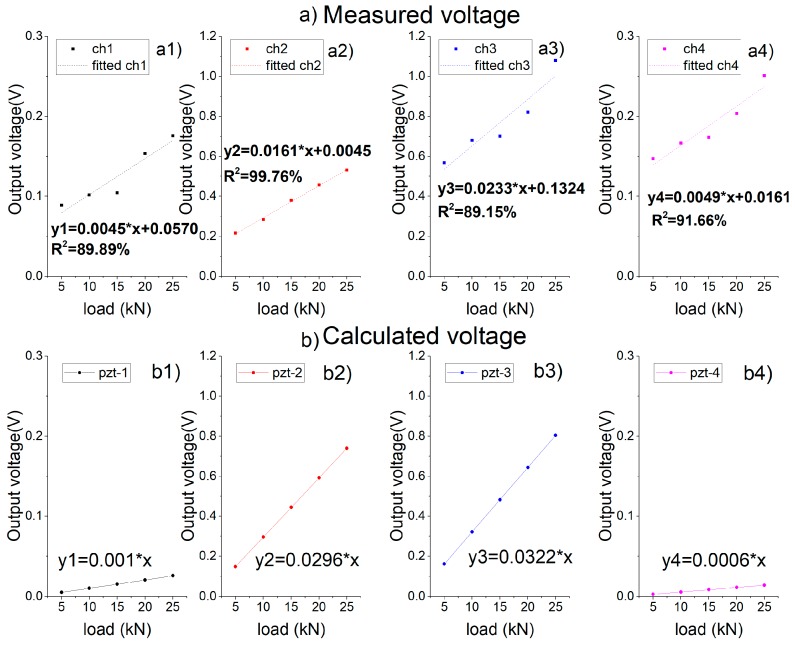
Comparison of measured and calculated values of each unit’s output at 10 Hz loading frequency. (**a**) measured results; (**b**) calculated results based on the stress value from the simulation.

Besides, the output performance of each unit has also been compared based on the experimental and calculated results, as shown in Figure 21. Although the measured slopes are not exactly the same as the calculated ones, the magnitudes of the slopes can be matched to each other for the same PZT patch, as well as the proportion of the slopes.

### 4.3. Simulation of Load Location Detecting

**Figure 22:**
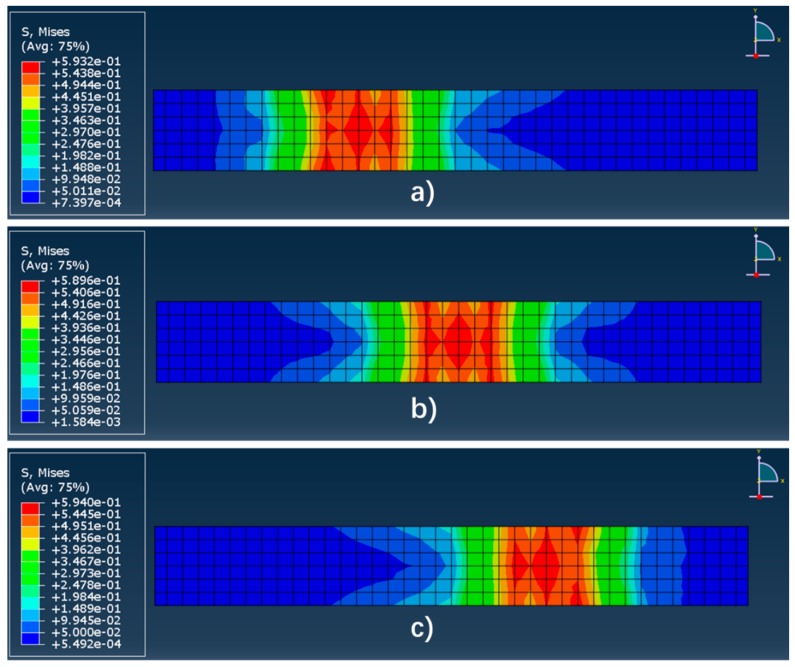
The stress distribution of the system surface under different loading position. (**a**) Load on the zone in the middle of 1# and 2# (Left); (**b**) load on the zone in the middle of 2# and 3# (Middle); (**c**) load on the zone in the middle of 3# and 4# (Right).

**Figure 23:**
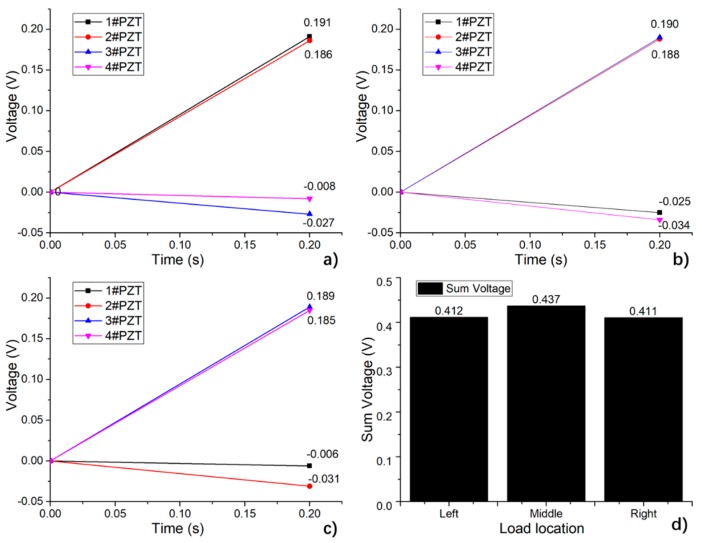
Output of the system changing with loading position. (**a**) Load on the zone in the middle of 1# and 2# (Left); (**b**) load on the zone in the middle of 2# and 3# (Middle); (**c**) load on the zone in the middle of 3# and 4# (Right); (**d**) comparison of the total output at different load locations.

By the above analysis, the finite element model used in this study can match the theoretical results well and coincide with the measured results. In order to further explore the application of the sensing system in the actual road, this section discusses the results of equivalent wheel load at different lateral positions. The simulated results are shown in Figure 22 and Figure 23.

According to Figure 22, when the non-uniform wheel load acts on different lateral positions of the system, the peak stresses on the surface of the system are 0.593 MPa (Left), 0.589 MPa (Middle), and 0.594 MPa (Right), respectively, sharing a similar stress distribution. The stress gradually decreases with the distance to the loading center increasing.

As shown in Figure 23a–c, the output voltage of a single piezoelectric unit is significantly affected by the loading position, and decreases as the distance between the load centers increases. Assumed the output voltage of 1#PZT to 4# PZT are *p1*, *p2*, *p3* and *p4* respectively, the load location identification can be expressed as:

(1) Put the output voltage in a vector, *p* = [ *p1*, *p2*, *p3*, *p4*];
(2) Sort the output voltage value of the patches in a descending order, with the corresponding number of PZT patch, [*psort*, *loc*] = sort (*p*, ‘descend’);
(3) Calculate the proportion of each value, *r* = [*psort(1)/psort(2)*, *psort(1)/psort(3)*, *psort(1)/psort(4)*];
(4) Deduce the location of the load center by the proportion results along with the vector of ‘*loc*’.

Taking the simulated voltage results shown in Figure 23a as an example, *p* = [0.191, 0.186, 0.027, 0.008], *psort* = [0.191, 0.186, 0.027, 0.008], *loc* = [1, 2, 3, 4], *r* = [1.0269, 7.0741, 23.8750], which can be concluded as the load center is almost at the middle point of 1#PZT to 2#PZT.

Thus, the load can be positioned according to the signal ratio of each piezoelectric unit. In this simulation, since the electric potential of the bottom surface of the piezoelectric units was set to zero, a negative voltage output occurred when the unit was strained. In practice, the measured voltage was the difference value of the upper and lower electrode of a piezoelectric unit, which is equivalent to the absolute value of the simulated voltage.

The sum of the absolute values of the output voltage of the piezoelectric unit is shown in Figure 23d. It can be seen that when a load with a certain peak value applies to different positions of the system, the total output deviation is less than 6%, and the deviation can be corrected by load positioning. Therefore, the design has good reliability when it serves as a location-identifying sensing system for a dynamic load.

### 4.4. Comparison with Other Weight-in-Motion (WIM) Systems

As introduced in the last part of Section 1, the designed piezoceramic sensing system is featured as low cost. The cost-effectiveness comparison of the proposed system and other WIM systems has been made from several aspects: the sensors, the measuring system, the labor for installation and the cost of lane closure [9,22]. Another two kinds of piezoelectric sensing systems have been compared to the proposed piezoceramic sensing system, as shown in Table 3. The sensor cost of those proposed are calculated from the fabricating process, and the measuring system contains the cost of a digital data acquisition machine and its accessories. The cost of installation and lane closure are reckoned according to the literature [38,39]. The results show that the proposed system has advantages both in unit cost of the sensor and the total cost of the system.

With the distributed sensing units, the proposed system can provide load location information by the internal relationship of the output voltage. For the piezo-quartz sensing system and the piezo-polymer cables, they are continuous and unable to detect the lateral location by a sensor.

Due to the overall size of the designed system, the cost of installation and lane closure is slightly higher than the piezo-polymer cables system, which will be lower after the size optimization. Without other deposition of the existing pavement, the costs of installation and disturbance to the traffic of the proposed system are much lower than those of the piezo-quartz systems. As analyzed in the above sections, the strength performance of the system is reliable, which means it can endure a complex overloading situation in the field.

## 5. Conclusions

In this paper, a distributed force sensing system based on piezoceramic unit is designed and fabricated. After theoretical analysis, indoor test and finite-element simulation, the following conclusions are obtained:

(1) The sensing system can measure the vertical load from 0.11 to 55.5 kN, with a stable structure and output performance. When the amplitude of the sinusoidal load increases, the total output of the system increases, and the linear correlation coefficient of the measured value is 94.6%. When the loading frequency is between 5–15 Hz, the total output of the system increases with the increase of the loading frequency, and the increasing rate decreases with the growing frequency; when the loading frequency varies from 15 to 19 Hz, the total output of the system fluctuates around a value of 1.305 V.
(2) The sensing system consists of several sensing units. The test shows that the output of each sensing unit decreases as the distance to the loading center increases. The simulation analysis further shows that the load position can be determined effectively according to the comparison of the output of different sensing units, and the location detection resolution is 120 mm. Meanwhile, when the load acts on different lateral positions of the system, the total output of the system changes no more than 6%, which can be corrected effectively according to the location-detection results.
(3) According to the structural design and test results, under the protection of the system package structure, the load on the sensing unit is 4.36% of the overall load of the system, indicating that the system can fully protect sensitive components and avoid premature failure.

In future, the system will be tested under higher loading frequencies (20–50 Hz) to expand its application. Field tests will be conducted to fully verify its feasibility under complex conditions; further research on promoting the location-detection resolution will be carried out as well. It can be seen from the above conclusions that the piezoceramic force sensor proposed in this study can be used as a new type of WIM system for pavement monitoring.

## Figures and Tables

**Table 1:** Main performance parameters of lead zirconate titanate (PZT-4).

Name	*Q_m_*	*R* (ohm)	*K_p_*	*C_p_* (nF)	$d_{33}\;(\mathrm{pC}/N)$
**Average**	180.38	19.54	0.59	3.785	369.9

Note: The quality factor (*Q_m_*), electric resistance (*R*) and electromechanical coupling coefficient (*K_p_*) are provided by the manufacturer, while the internal capacitance (*C_p_*) and piezoelectric strain constant ($d_{33}$) were measured in the lab.

**Table 2:** Model material parameters.

Layer	Elastic Modulus/MPa	Poisson’s Ratio	Density/(kg/m^3^)
**Fiber-reinforced nylon**	5900	0.34	1290
**PZT-4**	-	-	7500

**Table 3:** Cost comparison of the proposed system with the commercial sensing system.

Figure		Piezoceramic Sensing System	Piezo-Quartz Sensing System [38]	Piezo-Polymer Cables System [40]
Sensors	Unit cost ($)	540	6232	2077
	Quantity required per lane	8	4	2
	Total ($)	4320	24,928	4154
Measuring system ($)		6000	4072	9346
Installation ($)		7500	12,000	6500
Lane closure ($) [40]		11,000	20,000	10,000
Total cost ($)		28,820	61,000	30,000
